# The relationship between life satisfaction and bullying victimization: the mediating role of dysfunctional attitudes in gifted and typically developing students

**DOI:** 10.3389/fpsyg.2026.1825908

**Published:** 2026-08-11

**Authors:** Abdulkadir Muhammed Memduh Fakirullahoğlu

**Affiliations:** Istanbul Medipol University, School of Education, Department of Primary Education, Early Childhood Education Program, Istanbul, Türkiye

**Keywords:** gifted students, typically developing students, bullying victimization, life satisfaction, dysfunctional attitudes

## Abstract

Considering the prevalence of bullying in Türkiye, there are relatively few studies focusing on bullying victimization among gifted and typically developing students. This study examined the relationship between life satisfaction and bullying victimization among these two groups, considering the mediating role of dysfunctional attitudes. The participants comprised 486 students: 241 gifted students (99 female, 142 male; *M*_*age*_ = 11.29; *SD*_*age*_ = 1.35) and 245 typically developing students (118 female, 127 male; *M*_*age*_ = 11.68; *SD*_*age*_ = 1.36). Data were collected using the Satisfaction with Life Scale for Children (SWLS-C), the Dysfunctional Attitudes Scale for Children (8–14 years), and the Child–Adolescent Bullying Scale–9 Short Form. Data were analyzed using IBM SPSS Statistics 25 and AMOS, including multi-group structural equation modeling. The results revealed no significant difference in life satisfaction scores between gifted and typically developing students. However, compared with gifted students, typically developing students had higher scores for dysfunctional attitudes and bullying victimization. In addition, dysfunctional attitudes significantly mediated the association between life satisfaction and bullying victimization in both groups, and the structural paths did not differ significantly across groups. These findings highlight the importance of cognitive and socio-emotional factors in understanding bullying victimization among students.

## Introduction

Childhood and adolescence constitute critical periods characterized by intense physical, emotional, social, and cognitive development (Larsen and Luna, 2018). During these periods, there are numerous individual, familial, and environmental psychosocial factors associated with the development of children and adolescents (Demircioğlu and Yoldaş, 2019). Life satisfaction, as one of the fundamental indicators of psychological wellbeing, is a concept that reflects an individual’s level of satisfaction with their life and provides significant insights into their overall mental health (Demir et al., 2021; Pavot and Diener, 2009). Life satisfaction is closely related to adolescents’ psychosocial adjustment and their experiences of negative life events (Suldo and Huebner, 2004). Therefore, examining factors associated with life satisfaction is important for better understanding students’ psychological and social adjustment.

Although individuals in childhood and adolescence generally exhibit similar developmental characteristics, some may demonstrate extraordinary performance in specific domains compared to their peers (Renzulli, 2012). These students, who learn faster than their peers and stand out in cognitive, creative, leadership, artistic, or academic domains, are defined as gifted (Millî Eğitim Bakanlığı, 2026). Giftedness is a multidimensional concept that is not limited solely to high intelligence levels (Renzulli, 2016). According to Sternberg (2005), giftedness is the synthesis of wisdom, intelligence, and creativity. Similarly, Gagné (2004) describes this as a process in which innate potential is transformed into productivity through the interaction between personal and environmental factors.

Like their typically developing peers, gifted students may also experience various social and emotional problems (Akarsu and Mutlu, 2017). Students who struggle to cope with such problems are found to have lower levels of life satisfaction (Bergold et al., 2015; Gündoğdu and Toker, 2025). Although there are numerous studies in the literature on life satisfaction conducted with adults, the number of studies conducted with children and adolescents is quite limited (Aymerich et al., 2021; Ekşi et al., 2020; Marquez, 2024). Studies comparing life satisfaction between gifted and typically developing students appear to have been conducted on university students (Adam et al., 2025) and adolescents (Bergold et al., 2015). In these studies, no significant difference was found between the two groups in terms of life satisfaction levels. However, no study has been found that comparatively examines the life satisfaction levels of gifted and typically developing students in samples encompassing younger age groups. In this context, examining gifted and typically developing students within the same study may provide a clearer basis for understanding whether similar psychosocial and cognitive patterns are observed in both groups.

### Life satisfaction and bullying victimization

Life satisfaction refers to the cognitive appraisal an individual makes regarding their own quality of life (Diener et al., 1985). Accepted as the cognitive dimension of subjective wellbeing, this concept involves an individual’s positive or negative evaluation of their life conditions based on their self-determined criteria (Pavot and Diener, 2008). This evaluation is an important indicator for an individual’s overall quality of life and psychological wellbeing (Proctor et al., 2009). Therefore, life satisfaction in children and adolescents is associated with stressful life events and positive and negative affect (Wendt et al., 2019).

A review of the literature reveals that life satisfaction is associated with academic performance (Ng et al., 2015), as well as students’ psychosocial wellbeing, mental health, and potential future development (Sullanmaa et al., 2025). Reviewing 141 empirical studies that examined the relationships between life satisfaction and various constructs (emotional, social, and behavioral), Proctor et al. (2009) reported that low life satisfaction in youth is associated with behaviors such as aggression and violence. This finding suggests that lower life satisfaction may also be associated with negative peer-related experiences, such as bullying victimization.

Exposure to bullying in the school environment is a significant problem that can lead to negative outcomes, such as depression, low self-esteem, and a tendency toward criminal behavior in adulthood, in both the short and long term (Borgen et al., 2024; Olweus et al., 2019). Moreover, in a comprehensive meta-analysis examining the prevalence of bullying, data from 80 countries were analyzed, and it was reported that bullying occurs at a rate of approximately 35% globally (Modecki et al., 2014). The results indicate that approximately one in three students experiences bullying victimization and that bullying has become a prevalent and systemic part of school life. In this context, the Ministry of National Education (Millî Eğitim Bakanlığı, 2022) in Türkiye has also adopted a comprehensive approach in recent years, including educational programs and disciplinary processes aimed at combating peer bullying.

Bullying is a risk factor for gifted students as well as typically developing students (Peterson et al., 2009). Indeed, in their study conducted with primary and secondary school students, Škrabánková and Martínková (2018) determined that gifted students are in a riskier group compared to their peers and are exposed to different types of bullying. Therefore, research that comparatively examines the victimization of gifted students can contribute to a better understanding of the bullying cycle and the factors associated with this process.

Although bullying can be reduced through school-based interventions, it remains a serious problem involving multidimensional factors such as family, peer relationships, teacher attitudes, school environment, and the cultural context within the framework of the ecological systems approach (Espelage and Swearer, 2003). Exposure to bullying can lead to consequences such as emotional distress, low self-esteem, and even physical harm for the victim (Özbek-Baştuğ and Taneri, 2023). Such experiences may also be considered in relation to students’ distorted beliefs and dysfunctional attitudes. Indeed, according to cognitive theory, dysfunctional attitudes stem from rigid, irrational, and negative beliefs that the individual develops regarding themselves, others, and the world (Beck et al., 2023). From this perspective, dysfunctional attitudes can be considered a key psychological construct that may clarify the association between life satisfaction and bullying victimization.

### Dysfunctional attitudes as a mediator

Dysfunctional attitudes are cognitive structures that include irrational beliefs developed as a result of an individual’s interaction with their environment and that negatively affect their life (Beck, 2001). At the core of psychological problems lie distorted or dysfunctional thought patterns that negatively affect an individual’s mental health and behavior (Beck and Steer, 1987). These attitudes, which are difficult to change, can affect an individual’s emotions, thoughts, and behaviors.

Dysfunctional attitudes, the foundations of which are laid during childhood, strengthen alongside the cognitive development process and persist throughout life (D’Alessandro and Burton, 2006; Oral and Günlü, 2020). Furthermore, dysfunctional beliefs developed by the individual about themselves and others have negative effects on social skills and interpersonal relationships in adulthood (Cherrier et al., 2023). Thoughts such as “If I am always good, people will like me” or “Whenever I make a mistake, bad things happen” can be given as examples of these beliefs (Oral and Günlü, 2020). It is crucial to identify such attitudes, which are rooted in childhood and persist alongside cognitive development, at an early stage (Beck and Steer, 1987).

Because dysfunctional attitudes involve rigid and negative beliefs about the self, others, and the world (Beck, 2001; Beck et al., 2023), they may be relevant to both students’ evaluations of their lives and their peer-related experiences. Lower life satisfaction may be accompanied by stronger dysfunctional attitudes, and such attitudes may be associated with greater vulnerability to bullying victimization. Indeed, several studies have shown that dysfunctional attitudes are associated with bullying-related experiences (Kellij et al., 2022; Şahin and Sarı, 2010; Yan et al., 2022). From this perspective, dysfunctional attitudes may clarify the relationship between life satisfaction and bullying victimization.

In the present study, life satisfaction was theoretically positioned as the antecedent variable because it reflects students’ general cognitive evaluation of their lives and represents an important indicator of psychosocial wellbeing (Pavot and Diener, 2009). Dysfunctional attitudes were considered as the mediator within a cognitive-behavioral framework because they may be related to how students interpret themselves and peer-related experiences. Bullying victimization was examined as the outcome variable because the study focused on students’ self-reported victimization experiences in the school context. Accordingly, the proposed model examined whether dysfunctional attitudes mediated the relationship between life satisfaction and bullying victimization among gifted and typically developing students. However, given the cross-sectional design, alternative models are also plausible, and the proposed pathway should not be interpreted as evidence of temporal or causal ordering.

### The present study

Science and Art Centers (BİLSEM), which provide education for gifted students in Türkiye, are official institutions affiliated with the Ministry of National Education. In the 2025–2026 academic year, there were 106,415 gifted students across 362 BİLSEMs in 81 provinces (Millî Eğitim Bakanlığı, 2026). Given the number of students attending these centers, examining psychosocial variables such as life satisfaction, dysfunctional attitudes, and bullying victimization among gifted students is important for understanding their school-related adjustment and wellbeing. Examining the same mediation model among typically developing students may also provide a broader basis for understanding whether similar psychosocial and cognitive patterns are observed in both student groups.

Based on the finding that bullying behaviors peak during middle school and tend to decline thereafter (Goldbaum et al., 2003), this study was planned to cover the upper levels of elementary school and the middle school period. Accordingly, this study examined the relationship between life satisfaction and bullying victimization among gifted and typically developing students, as well as the mediating role of dysfunctional attitudes in this relationship. The mediation model was tested using multi-group structural equation modeling to examine the pattern of direct and indirect associations across the two student groups.

## Materials and methods

### Participants

The participants were 486 gifted and typically developing students studying at the elementary and middle school levels. Of these participants, 241 were gifted students attending Science and Art Centers, and 245 were typically developing students attending public schools. In the present study, the term typically developing students refers to students who attended public schools, had not been identified as gifted through the national identification process, and were not enrolled in Science and Art Centers.

Gifted students were selected through convenience sampling from students officially enrolled in BİLSEM. In Türkiye, admission to BİLSEM is based on the national identification process conducted by the Ministry of National Education. In this process, students who qualify in the initial screening stage proceed to an individual assessment stage, in which standardized intelligence or ability tests such as the Wechsler Nonverbal Scale of Ability (WNV) and the Anadolu-Sak Intelligence Scale (ASIS) may be administered.

In the present study, giftedness was operationally defined as being officially enrolled in BİLSEM at the time of data collection. No additional intelligence, ability, teacher nomination, or academic achievement criteria were applied by the researcher for re-identification purposes. Students in the typically developing group were not identified as gifted, were not enrolled in BİLSEM, and were not receiving special education services according to school records. No separate clinical screening was conducted by the researcher for developmental, emotional, behavioral, or learning difficulties.

### Typically developing students

The typically developing group comprised 245 students, of whom 118 were female (48.2%) and 127 were male (51.8%). The mean age of the students is 11.68 (SD = 1.36). Regarding grade level distribution, 48 students are in 4th grade (19.6%), 52 in 5th grade (21.2%), 51 in 6th grade (20.8%), 51 in 7th grade (20.8%), and 43 in 8th grade (17.6%). In terms of family structure, 211 students (86.1%) reported coming from two-parent families, while 34 students (13.9%) reported that their parents were separated or divorced. The educational levels of the parents are as follows: for fathers, 24.5% have less than a high school diploma, 40.8% are high school graduates, and 34.7% are university graduates. For mothers, 29.8% have less than a high school diploma, 36.7% are high school graduates, and 33.5% are university graduates. Regarding perceived socioeconomic status, 36.1% of the students reported a low level, 46.9% a medium level, and 17.1% a high level.

### Gifted students

The second participant group included in this study consists of 241 gifted students studying at Science and Art Centers (BİLSEM) in Türkiye. Of these participants, 99 are female (41.1%) and 142 are male (58.9%). The mean age of the students is 11.29 (SD = 1.35). In terms of grade level distribution, 64 students are in 4th grade (26.6%), 48 in 5th grade (19.9%), 61 in 6th grade (25.3%), 43 in 7th grade (17.8%), and 25 in 8th grade (10.4%). Regarding family structure, 220 students (91.3%) reported coming from two-parent families, while 21 students (8.7%) reported that their parents were separated or divorced. The educational levels of the parents are as follows: for fathers, 9.9% have less than a high school diploma, 24.9% are high school graduates, and 65.1% are university graduates. For mothers, 10.4% have less than a high school diploma, 25.7% are high school graduates, and 63.9% are university graduates. Regarding perceived socioeconomic status, 31.1% of the students reported a low level, 56.4% a medium level, and 12.4% a high level.

### Measures

#### Satisfaction with Life Scale for Children

The Satisfaction with Life Scale for Children, developed by Gadermann et al. (2010), was adapted into Turkish by Altay and Ekşi (2018). In the adaptation study, the fit index values obtained from the confirmatory factor analysis method (χ^2^/df = 0.80; RMSEA = 0.01; GFI = 0.99; AGFI = 0.98; CFI = 1.00) showed that the structure of the scale was appropriate. In the adaptation study, the Cronbach’s alpha internal consistency coefficient of the scale was found to be 0.79, whereas it was 0.76 in the current study. The scale has a single-dimensional structure, consists of five items, and is rated on a five-point Likert-type scale ranging from 1 (strongly disagree) to 5 (strongly agree). A sample item is “I am happy with my life.” Higher total scores indicate a higher level of life satisfaction. In the present study, total scores were used in the analyses.

#### Dysfunctional attitudes scale for children (8–14 years)

The scale, developed by D’Alessandro and Burton (2006), was adapted into Turkish by Oral and Günlü (2020). The researchers conducted the adaptation study using confirmatory factor analysis, and the fit indices obtained from the analysis (χ^2^/df = 1.58; RMSEA = 0.03; SRMR = 0.04; GFI = 0.93; AGFI = 0.92; CFI = 0.91) indicated that the scale structure was acceptable. While the Cronbach’s alpha internal consistency coefficient was 0.82 in the adaptation study, it was.83 in the current study. The scale has a single-dimensional structure and consists of 22 items rated on a five-point Likert-type scale ranging from 1 (strongly disagree) to 5 (strongly agree). A sample item is “I should be better than other children.” Total scores range from 22 to 110, with higher scores indicating higher levels of dysfunctional attitudes. In the present study, total scores were used in the analyses.

#### Child-Adolescent Bullying Scale-9 Short Form

The Child-Adolescent Bullying Scale–9 Short Form was developed by Vessey et al. (2019), and its Turkish adaptation was conducted by Kırıcı and Ekşi (2022). The fit index values obtained from confirmatory factor analysis (χ^2^/df = 3.947; RMSEA = 0.08; SRMR = 0.04; GFI = 0.93; TLI = 0.92; CFI = 0.94) indicated that the scale structure was acceptable. In the adaptation study, the Cronbach’s alpha internal consistency coefficient of the scale was 0.88, whereas it was 0.85 in the current study. The scale has a single-factor structure and consists of nine items rated on a four-point Likert-type scale. A sample item is “Children share mean or hurtful messages, comments, or photos about me online.” Total scores range from 9 to 36, with higher total scores indicating higher levels of bullying victimization. In the present study, total scores were used in the analyses (Kırıcı and Ekşi, 2022; Vessey et al., 2019).

### Procedure

Before data collection, ethical approval was obtained from the Istanbul University-Cerrahpaşa Social and Humanities Research Ethics Committee (Decision No. 2025/261; April 18, 2025). Following approval, data were collected from two different Science and Art Centers (BİLSEM) and two different public schools. Before data collection, participants were informed about the purpose and process of the research, and parental consent forms were obtained. All participants volunteered to participate in the research. Data were collected by administering the self-report scales in person during school hours under the supervision of the researcher.

### Data analysis

Within the scope of the research, 250 gifted students attending Science and Art Centers and 260 typically developing students attending two public schools were reached. Prior to the main analyses, outlier analysis and normality tests were performed. Standardized z-scores were computed separately within each group for life satisfaction, dysfunctional attitudes, and bullying victimization. As a result of the outlier analysis, data from 9 gifted and 15 typically developing students whose z-scores had an absolute value greater than 3 were excluded from the analysis (Field, 2009). As a sensitivity analysis, the group comparisons were also examined using the full dataset before outlier exclusion, and the pattern of findings was substantively similar. Therefore, all main analyses were reported using the final dataset after outlier exclusion.

For the assessment of data distribution, the criterion that skewness and kurtosis values should be within the range of ± 1.5 was adopted (Tabachnick and Fidell, 2013). The obtained values indicated that the distributions of the variables in both groups were normal (see Tables 1, 2). During the analysis process, descriptive statistics were calculated, and Pearson correlation analysis was applied to determine the relationships between the variables. An independent-samples *t*-test was conducted to determine whether there were significant differences between the two groups in terms of life satisfaction, dysfunctional attitudes, and bullying victimization scores.

**TABLE 1:** Descriptive statistics and correlations of the gifted students’ variables.

Variable	*M*	SD	Skewness	Kurtosis	1	2
1. Life satisfaction	18.10	4.72	–0.57	–0.29	1	
2. Dysfunctional attitudes	49.22	14.08	0.15	–0.35	–0.29^***^	1
3. Bullying victimization	15.09	5.38	0.74	–0.02	–0.39^***^	0.49^***^

*N* = 241,

^***^p < 0.001.

**TABLE 2:** Descriptive statistics and correlations among the study variables for typically developing students.

Variable	*M*	SD	Skewness	Kurtosis	1	2
1. Life satisfaction	17.71	4.54	–0.32	–0.41	1	
2. Dysfunctional attitudes	55.91	13.11	0.24	–0.33	–0.17^**^	1
3. Bullying victimization	16.62	5.35	0.47	–0.55	–0.31^***^	0.30^***^

*N* = 245,

^**^p < 0.01,

^***^p < 0.001.

After the preliminary analyses, the measurement and structural models were tested using AMOS. First, the overall measurement model was examined. Then, measurement invariance across gifted and typically developing students was tested using multi-group confirmatory factor analysis. Configural, metric, scalar, and strict invariance models were tested sequentially. Model comparisons were evaluated using χ^2^ difference values and changes in CFI. Following the measurement invariance analyses, a multi-group structural equation model was tested to examine the direct and indirect associations among life satisfaction, dysfunctional attitudes, and bullying victimization across the two groups. Structural path equality across groups was evaluated by comparing constrained and unconstrained models. Indirect effects were tested using bias-corrected bootstrap confidence intervals based on 2,000 resamples. IBM SPSS Statistics 25 was used for descriptive statistics, correlations, and group comparisons, whereas AMOS was used for confirmatory factor analysis, measurement invariance testing, and multi-group structural equation modeling.

## Results

### Preliminary results

Descriptive statistics for variables related to gifted students are presented in Table 1.

As seen in Table 1, life satisfaction was significantly and negatively associated with dysfunctional attitudes (*r* = −0.29, *p* < 0.001) and bullying victimization (*r* = −0.39, *p* < 0.001). In addition, dysfunctional attitudes were significantly and positively associated with bullying victimization (*r* = 0.49, *p* < 0.001).

As seen in Table 2, life satisfaction was significantly and negatively associated with dysfunctional attitudes (*r* = −0.17, *p* = 0.007) and bullying victimization (*r* = −0.31, *p* < 0.001). In addition, dysfunctional attitudes were significantly and positively associated with bullying victimization (*r* = 0.30, *p* < 0.001).

An independent-samples *t*-test was conducted to examine differences between gifted and typically developing students in life satisfaction, dysfunctional attitudes, and bullying victimization (see Table 3).

**TABLE 3:** Differences in life satisfaction, dysfunctional attitudes, and bullying victimization.

Variable	Gifted *M* (SD)	Typically developing *M* (SD)	*t*	*p*	Cohen’s *d*	95% CI
Life satisfaction	18.10 (4.72)	17.71 (4.54)	0.936	0.350	0.08	(–0.09, 0.26)
Dysfunctional attitudes	49.22 (14.08)	55.91 (13.11)	–5.423*	0.001	–0.49	(–0.67, –0.31)
Bullying victimization	15.09 (5.38)	16.62 (5.35)	–3.132*	0.002	–0.29	(–0.46, –0.11)

Standard deviations are presented in parentheses. CI, confidence interval for Cohen’s *d*.

*p < 0.05.

The results indicated no significant difference in life satisfaction between gifted students (*M* = 18.10, *SD* = 4.72) and typically developing students (*M* = 17.71, *SD* = 4.54), *t*(484) = 0.936, *p* = 0.350. However, gifted students (*M* = 49.22, *SD* = 14.08) reported significantly lower levels of dysfunctional attitudes than typically developing students (*M* = 55.91, *SD* = 13.11), *t*(484) = -5.423, *p* < 0.001. Likewise, gifted students (*M* = 15.09, *SD* = 5.38) reported significantly lower levels of bullying victimization than typically developing students (*M* = 16.62, *SD* = 5.35), *t*(484) = -3.132, *p* = 0.002.

### Measurement invariance

Prior to conducting group comparisons, measurement invariance of the three scales was examined using multi-group confirmatory factor analysis (MGCFA) in AMOS. A sequence of increasingly constrained models was tested: configural, metric, scalar, and strict invariance. Before the multi-group confirmatory model, the overall measurement model was also tested without grouping (χ^2^/df = 2.259, RMSEA = 0.051, GFI = 0.861, SRMR = 0.058). The multi-group CFA results are presented in Table 4.

**TABLE 4:** Results of multi-group confirmatory factor analysis: tests of measurement invariance across gifted and typically developing students.

Model	χ ^2^	df	χ ^2^/df	CFI	RMSEA	Δχ ^2^	Δ df	Δ CFI
M1: Configural	2054.08	1,174	1.750	0.831	0.039	–	–	–
M2: Metric	2152.94	1,207	1.784	0.818	0.040	98.87*	33	–0.013
M3: Scalar	2161.57	1,213	1.782	0.817	0.040	8.63	6	–0.001
M4: Strict	2279.99	1,253	1.820	0.802	0.041	118.42*	40	–0.015

Δχ^2^ and ΔCFI values reflect changes relative to the preceding model.

*p < 0.05.

The configural model (M1), which imposed no equality constraints across groups, served as the baseline and demonstrated acceptable fit (χ^2^ = 2054.08, df = 1174, χ^2^/df = 1.750, CFI = 0.831, RMSEA = 0.039), confirming that the same factor structure held in both groups. Constraining the factor loadings to be equal across groups (M2: metric model) resulted in a marginal decrease in fit (ΔCFI = -0.013). Although this value slightly exceeds the commonly recommended threshold of -0.010 (Cheung and Rensvold, 2002), chi-square difference tests are known to be sensitive to sample size, and the absolute change in RMSEA remained negligible (ΔRMSEA = 0.001). The metric model was therefore considered to reflect at least partial metric invariance, sufficient for meaningful comparison of structural relationships. Importantly, the transition from the metric to the scalar model (M3), which additionally constrained the item intercepts to equality, produced virtually no change in fit (ΔCFI = -0.001, Δχ^2^(6) = 8.63, *p* > 0.05), indicating that scalar invariance was fully supported. The strict invariance model (M4), which further constrained residual variances, resulted in a notable decline in fit (ΔCFI = –0.015) and was therefore not retained. Overall, the results supported the comparison of latent means and structural paths across gifted and typically developing students.

### Multi-group structural model results

After measurement invariance was examined, a multi-group structural equation model was tested to compare the structural paths across gifted and typically developing students. The model comparison results are presented in Table 5.

**TABLE 5:** Fit indices for the multi-group structural equation model across gifted and typically developing students.

Model	χ ^2^	df	χ ^2^/df	CFI	RMSEA	Δχ ^2^	Δ df	Δ CFI
M1: Unconstrained	2029.08	1,172	1.731	0.835	0.039	–	–	–
M2: Measurement weights	2129.74	1,205	1.767	0.822	0.040	100.66*	33	–0.013
M3: Structural weights	2136.38	1,208	1.769	0.821	0.040	6.63	3	–0.001
M4: Structural covariances	2137.49	1,209	1.768	0.821	0.040	1.11	1	0.000
M5: Structural residuals	2138.23	1,211	1.766	0.822	0.040	0.74	2	0.000
M6: Measurement residuals	2234.31	1,247	1.792	0.810	0.040	96.08*	36	–0.012

Δχ^2^ and ΔCFI values reflect change relative to the preceding model. The baseline model (M1) incorporated five modification indices to improve fit (correlated residuals between items e1–e2, e1–e18, e10–e19, e11–e12, and e30–e34), applied consistently across both groups.

*p < 0.05.

**FIGURE 1:**
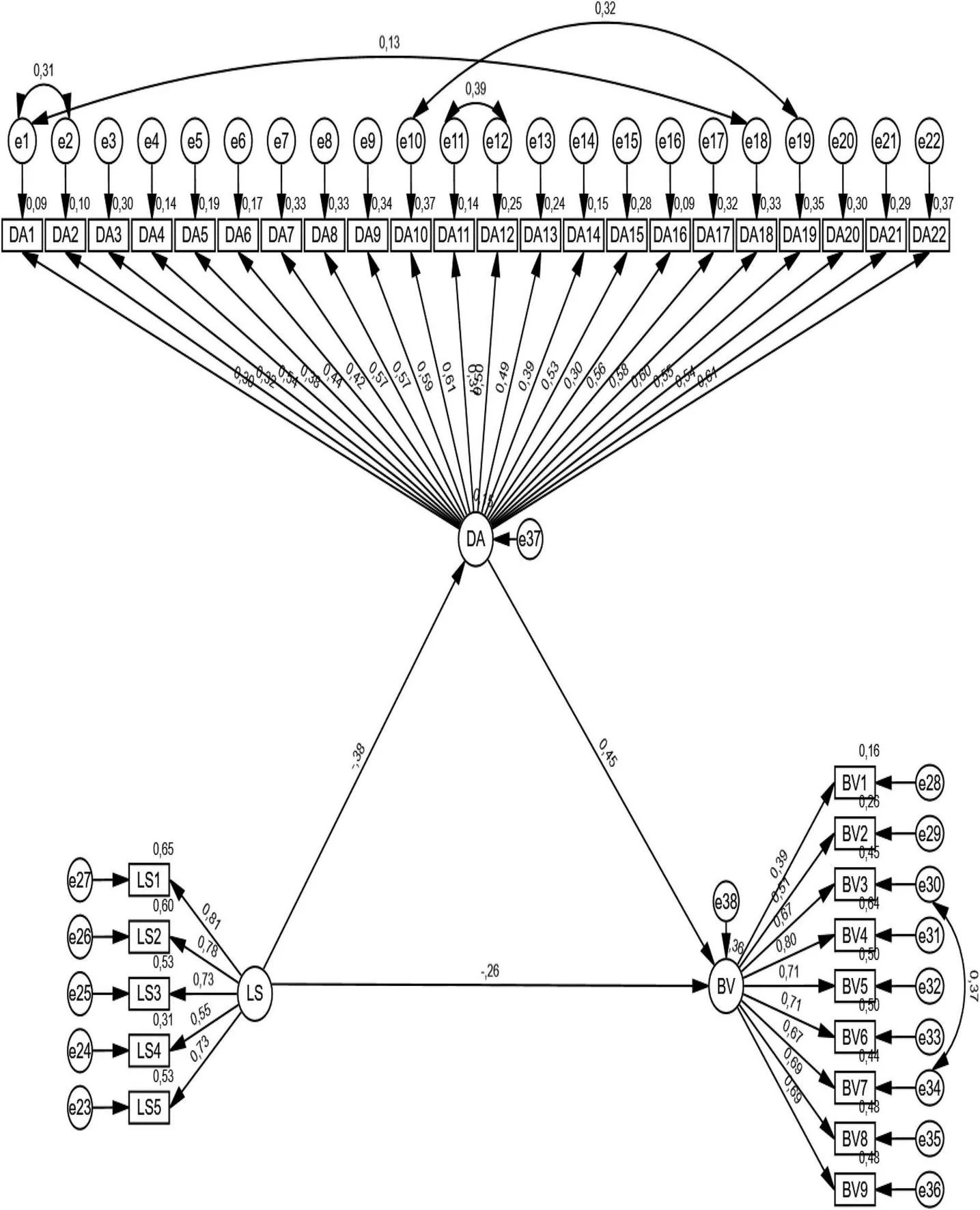
Structural equation model for gifted students. Standardized path coefficients are presented. LS, life satisfaction; DA, dysfunctional attitudes; BV, bullying victimization.

**FIGURE 2:**
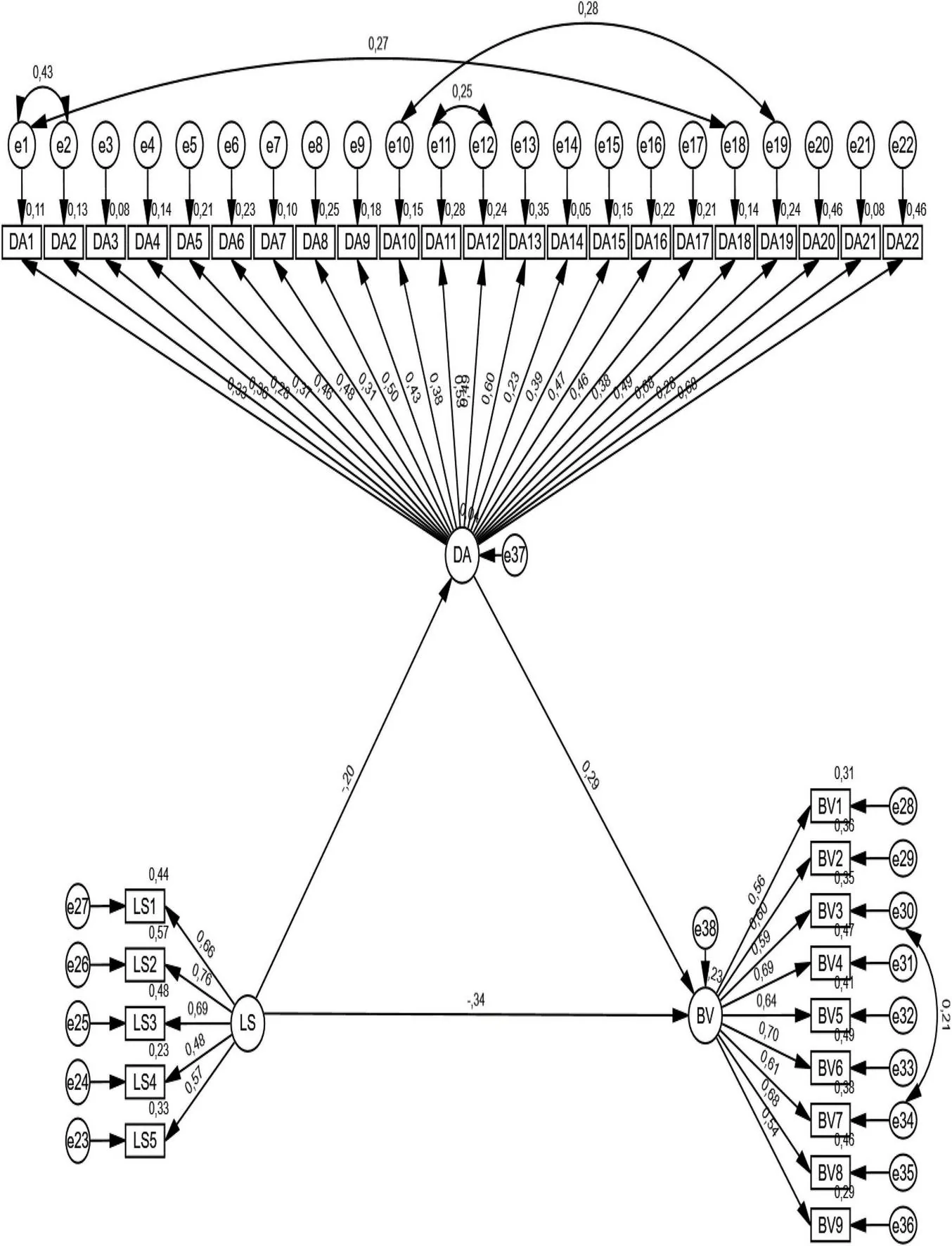
Structural equation model for typically developing students. Standardized path coefficients are presented. LS, life satisfaction; DA, dysfunctional attitudes; BV, bullying victimization.

As seen in Table 5, constraining the structural paths to equality across groups did not significantly worsen model fit, Δχ^2^(3) = 6.63, *p* = 0.085, ΔCFI = -0.001. This finding indicates that the structural paths in the mediation model did not differ significantly between gifted and typically developing students. The standardized structural models for gifted and typically developing students are presented in Figures 1, 2.

The standardized path coefficients, indirect effects, and bootstrap confidence intervals obtained from the multi-group SEM analysis are presented in Table 6.

**TABLE 6:** Standardized path coefficients, indirect effects, and bootstrap confidence intervals from multi-group structural equation modeling.

Path	Gifted				Typically developing students			
	β	*SE*	*p*	95% CI	β	*SE*	*p*	95% CI
Direct effects								
LS → DA	–0.384	0.038	< 0.001	(–0.522, –0.232)	–0.204	0.043	0.018	(–0.374, –0.033)
DA → BV	0.450	0.117	0.001	(0.289, 0.589)	0.285	0.108	0.003	(0.122, 0.447)
LS → BV (direct)	–0.263	0.023	0.003	(–0.434, –0.107)	–0.335	0.049	0.001	(–0.462, –0.196)
Indirect effect (LS → DA → BV)								
DA as mediator	–0.173	0.042	< 0.001	(–0.268, –0.103)	–0.058	0.030	0.009	(–0.138, –0.013)

β, standardized path coefficient; SE, standard error from maximum likelihood estimation; 95% CI, bias-corrected bootstrap confidence interval (2,000 resamples) for total effects from the unconstrained model. LS, life satisfaction; DA, dysfunctional attitudes; BV, bullying victimization. The indirect effect (β) is computed as the product of the two standardized path coefficients (a × b); its significance was evaluated via bias-corrected bootstrap two-tailed significance test (*p* = 0.001 for both groups). Formal test of structural path equality across groups: Δχ^2^(3) = 6.63, *p* = 0.085, ΔCFI = -0.001, indicating that the structural paths did not differ significantly between groups.

As seen in Table 6, life satisfaction had a significant and negative direct effect on dysfunctional attitudes among gifted students [β = –0.384, *p* < 0.001, 95% CI (–0.522, –0.232)]. Dysfunctional attitudes had a significant and positive direct effect on bullying victimization [β = 0.450, *p* = 0.001, 95% CI (0.289, 0.589)]. In addition, the direct effect of life satisfaction on bullying victimization was significant and negative [β = –0.263, *p* = 0.003, 95% CI (–0.434, –0.107)]. In the gifted group, life satisfaction and dysfunctional attitudes explained 36.3% of the variance in bullying victimization (*R*^2^ = 0.363).

Among typically developing students, life satisfaction also had a significant and negative direct effect on dysfunctional attitudes [β = –0.204, *p* = 0.018, 95% CI (–0.374, –0.033)]. Dysfunctional attitudes had a significant and positive direct effect on bullying victimization [β = 0.285, *p* = 0.003, 95% CI (0.122, 0.447)]. The direct effect of life satisfaction on bullying victimization was also significant and negative [β = –0.335, *p* = 0.001, 95% CI (–0.462, –0.196)]. In the typically developing group, life satisfaction and dysfunctional attitudes explained 23.3% of the variance in bullying victimization (*R*^2^ = 0.233).

Regarding the indirect effects, the indirect effect of life satisfaction on bullying victimization through dysfunctional attitudes was statistically significant for both gifted students [β = –0.173, *p* < 0.001, 95% CI (–0.268, –0.103)] and typically developing students [β = –0.058, *p* = 0.009, 95% CI (–0.138, –0.013)]. Thus, dysfunctional attitudes partially mediated the association between life satisfaction and bullying victimization in both groups. Although the magnitude of the indirect effect was larger among gifted students (β = -0.173) than among typically developing students (β = –0.058), the equality restriction test showed that the structural pathways did not differ significantly between the groups, Δχ^2^(3) = 6.63, *p* = 0.085, ΔCFI = –0.001. These findings indicate that, despite small differences in effect size, the underlying mediating mechanism operated similarly in both groups.

## Discussion

This study examined the association between life satisfaction and bullying victimization among gifted and typically developing students, as well as the mediating role of dysfunctional attitudes in this association. The findings revealed no significant difference in life satisfaction scores between the two groups. However, compared with gifted students, typically developing students had significantly higher scores for dysfunctional attitudes and bullying victimization. In addition, dysfunctional attitudes significantly mediated the association between life satisfaction and bullying victimization in both groups. The multi-group SEM results further indicated that the structural paths did not differ significantly across groups.

The first finding of this study did not indicate a significant difference in life satisfaction scores between gifted and typically developing students. Consistent with the present study, previous research has reported comparable levels of life satisfaction among gifted and typically developing students (Adam et al., 2025; Bergold et al., 2015). Life satisfaction remains an important consideration for all students; for gifted students, it may also be relevant to the process of actualizing their potential (Görgöz and Aktan, 2023). However, the present finding suggests that life satisfaction should not be interpreted solely in relation to giftedness in the present sample. Rather, this result supports the view that life satisfaction is a multidimensional and complex construct. Therefore, guidance practices that support students’ wellbeing may be relevant for both gifted and typically developing students (Huebner et al., 2004).

The findings further revealed that gifted students had significantly lower scores for dysfunctional attitudes than their typically developing peers. One possible interpretation of this finding is that it may be related to the cognitive and metacognitive characteristics often associated with giftedness. Indeed, the literature indicates that gifted students may demonstrate more advanced cognitive flexibility, self-regulation, and abstract thinking skills, which may be associated with a greater capacity to question negative and rigid beliefs (Rinn and Majority, 2018). In addition, frequent and early experiences of success among gifted students may be relevant to the development of less rigid self-related beliefs within the framework of Beck’s cognitive model (Beck, 2001). Nevertheless, this interpretation should be considered cautiously, as individual and environmental factors may also shape dysfunctional attitudes. To date, no study has been found that directly compares cognitive distortions or dysfunctional attitudes between gifted and typically developing students. However, research conducted with typically developing students has shown that dysfunctional attitudes are associated with variables such as perfectionism and perceived social support (Yang et al., 2025), bullying tendencies (Şahin and Sarı, 2010), and depression and anxiety (Yang et al., 2021).

The third finding of the study indicated that gifted students had significantly lower bullying victimization scores than their typically developing peers. Higher-level cognitive functions may be associated with the use of more adaptive coping strategies when encountering stressful situations such as bullying (Sameroff and Rosenblum, 2006). However, studies comparing gifted and typically developing students have yielded inconsistent results. While some studies suggest that gifted and typically developing students experience similar rates of victimization (Mitchell, 2011; Peters and Bain, 2011), González-Cabrera et al. (2023) reported that gifted students had a higher probability of being victimized (41.1% vs. 21.8%). Similarly, Ogurlu and Sarıçam (2018) stated that gifted students’ exposure to bullying was significantly higher than that of their typically developing peers. These findings suggest that bullying is a multidimensional and complex phenomenon, and that individual and contextual differences may be relevant to understanding group-based variations in bullying victimization.

Life satisfaction was negatively and significantly associated with bullying victimization among typically developing students. Individuals with higher life satisfaction generally report more positive self-perception, stronger social support networks, and more effective coping skills (Katsantonis et al., 2024; Yang et al., 2021). These characteristics may provide a framework for interpreting the negative association between life satisfaction and bullying victimization. This research finding is consistent with numerous studies revealing a negative correlation between life satisfaction and bullying victimization (Flaspohler et al., 2009; Navarro et al., 2015; Varela et al., 2019; Yang et al., 2021; Yavaş, 2021; You et al., 2008). Indeed, Cingöz (2025), in a study based on PISA 2022 Türkiye data, reported that students with high life satisfaction had significantly lower rates of exposure to bullying.

Similar to typically developing students, life satisfaction was negatively and significantly associated with bullying victimization among gifted students. This finding suggests that life satisfaction is not only relevant to general wellbeing among gifted students but may also be associated with their peer victimization experiences. Furthermore, the fact that this relationship emerges similarly in both groups indicates that the association between life satisfaction and bullying victimization was observed in both groups. Although no study in the literature has examined the link between traditional bullying victimization and life satisfaction in gifted students, it has been determined that those victimized by cyberbullying report significantly lower life satisfaction scores (González-Cabrera et al., 2019). While no national study has focused directly on life satisfaction, Kara and Aslan (2025) reported that cyber-victimization was negatively associated with the subjective wellbeing of gifted youth, and that this association was moderated by self-esteem and social support. In this context, life satisfaction may be considered a relevant psychosocial correlate of bullying victimization among gifted students.

Examination of the study’s primary mediation finding revealed that dysfunctional attitudes significantly mediated the association between life satisfaction and bullying victimization in both groups. Higher levels of dysfunctional attitudes may be associated with more persistent negative thought patterns and greater difficulty in interpreting social conflicts adaptively (Beck, 1979). In this regard, dysfunctional attitudes may provide a cognitive framework for understanding why lower life satisfaction is associated with higher bullying victimization. Previous research supports this association; for instance, Kellij et al. (2022) found a significant relationship between bullying victimization and social-cognitive distortions as well as negative cognitive structures. Similarly, Şahin and Sarı (2010) demonstrated a significant correlation between cognitive distortions and cyberbullying victimization. In a longitudinal study, Yan et al. (2022) demonstrated the mediating role of dysfunctional cognitions in the association between bullying and psychological outcomes, such as depression and anxiety, among children aged 8–11. In the present study, although the indirect effect was numerically larger among gifted students, the multi-group SEM results indicated that the structural pathways did not differ significantly between the groups. Taken together, these findings suggest that dysfunctional attitudes may represent an important cognitive dimension in understanding bullying victimization within the school context.

## Limitations and future research

This study contributes to the literature on bullying victimization among gifted and typically developing students at the elementary and middle school levels. However, several limitations should be considered when interpreting the findings. First, the study employed a cross-sectional design based on self-report measures. Therefore, causal, predictive, or temporal conclusions cannot be drawn, and the mediation results should be interpreted as statistical associations rather than evidence of causal mechanisms. Second, the data were obtained from students attending elementary schools, middle schools, and Science and Art Centers (BİLSEM) in Istanbul. Gifted students were recruited from Science and Art Centers, whereas typically developing students were recruited from public schools. Therefore, institutional context, educational opportunities, and selection-related factors may have influenced the observed group differences. In addition, potential covariates such as family support, peer relationships, school climate, parental education, and socio-economic status were not controlled for in the analyses. Finally, cyberbullying was not assessed within the scope of this study. Including cyberbullying in future research may contribute to a more comprehensive understanding of different forms of bullying victimization among gifted and typically developing students.

## Conclusion

The overall results of the study indicate that life satisfaction, dysfunctional attitudes, and bullying victimization are significantly interrelated among gifted and typically developing students. Dysfunctional attitudes significantly mediated the association between life satisfaction and bullying victimization in both groups. Although no significant difference was found in life satisfaction scores between gifted and typically developing students, typically developing students had higher scores for dysfunctional attitudes and bullying victimization. These findings highlight the relevance of socio-emotional and cognitive factors in understanding bullying victimization among students. In particular, dysfunctional attitudes may represent an important cognitive dimension that should be considered in future school-based research and guidance practices.

## Data Availability

The raw data supporting the conclusions of this article will be made available by the authors, without undue reservation.
